# Viral Threats to Fruit and Vegetable Crops in the Caribbean

**DOI:** 10.3390/v16040603

**Published:** 2024-04-13

**Authors:** Paula Tennant, Sephra Rampersad, Angela Alleyne, Lloyd Johnson, Deiondra Tai, Icolyn Amarakoon, Marcia Roye, Patrice Pitter, Peta-Gaye Chang, Lisa Myers Morgan

**Affiliations:** 1 Department of Life Sciences, The University of the West Indies, Mona, St. Andrew JMAAW07, Jamaica; lloydj@torontomu.ca; 2 Biotechnology Centre, The University of the West Indies, Mona, St. Andrew JMAAW07, Jamaica; deiondra.robinson02@uwimona.edu.jm (D.T.); macia.roye@uwimona.edu.jm (M.R.); pitterpat099@gmail.com (P.P.); 3 Department of Life Sciences, The University of the West Indies, St. Augustine 999183, Trinidad and Tobago; sephra.rampersad@sta.uwi.edu; 4 Department of Biological and Chemical Sciences, The University of the West Indies, Cave Hill, Bridgetown BB11000, Barbados; angela.alleyne@cavehill.uwi.edu; 5 Department of Basic Medical Sciences, Biochemistry Section, Faculty of Medical Sciences Teaching and Research Complex, The University of the West Indies, Mona, St. Andrew JMAAW07, Jamaica; icolyn.amarkoon@uwimona.edu.jm; 6 Ministry of Agriculture, Bodles Research Station, Old Harbour, St. Catherine JMACE18, Jamaica; petagaye.chang@moa.gov.jm (P.-G.C.); lisa.myersmorgan@moa.gov.jm (L.M.M.)

**Keywords:** biosafety, biosecurity, detection, developing countries, diversity, epidemiology, integrated disease management, transmission

## Abstract

Viruses pose major global challenges to crop production as infections reduce the yield and quality of harvested products, hinder germplasm exchange, increase financial inputs, and threaten food security. Small island or archipelago habitat conditions such as those in the Caribbean are particularly susceptible as the region is characterized by high rainfall and uniform, warm temperatures throughout the year. Moreover, Caribbean islands are continuously exposed to disease risks because of their location at the intersection of transcontinental trade between North and South America and their role as central hubs for regional and global agricultural commodity trade. This review provides a summary of virus disease epidemics that originated in the Caribbean and those that were introduced and spread throughout the islands. Epidemic-associated factors that impact disease development are also discussed. Understanding virus disease epidemiology, adoption of new diagnostic technologies, implementation of biosafety protocols, and widespread acceptance of biotechnology solutions to counter the effects of cultivar susceptibility remain important challenges to the region. Effective integrated disease management requires a comprehensive approach that should include upgraded phytosanitary measures and continuous surveillance with rapid and appropriate responses.

## 1. Introduction

Despite efforts in plant health management, viral diseases continue to threaten the productivity and sustainability of agriculture. These diseases are caused by newly emerging and recurring pathogens that have expanded into alternate hosts or geographical locations or undergone changes in virulence [1,2]. International trade [3,4], along with agricultural expansion [5,6], changes in agricultural practices [7,8,9] and genetic changes in virus species [10,11], are some of the underlying causes of emergence. Mono-cropping, characterized by high plant density and uniform plant genetics, also creates ideal conditions for disease emergence [12], as well as weeds that serve as virus reservoirs and contribute to virus diversification and evolution [13,14,15]. Furthermore, there is growing evidence that supports climate change as one of the more important drivers of virus disease outbreaks [16,17,18]. The ecology and dynamics of emerging and re-emerging viruses are complex and multifaceted, warranting early identification together with continuous monitoring and control, both regionally and globally [19].

The Caribbean region is located between the southernmost parts of Florida and northeastern Venezuela; this region consists of more than 7,000 islands subdivided into three groups: The Bahamas; The Greater Antilles which consists of the larger islands e.g., Cuba, Hispaniola, Jamaica, and Puerto Rico; and The Lesser Antilles, which consists of smaller islands, e.g., Barbados and Trinidad and Tobago [20]. The climate of the region is characterized by temperatures that remain consistently high year-round (24–27 °C to 29–35 °C) and relative humidity between 70–90%, and there are two main seasons characterized by differences in temperature and rainfall [21,22]. The region has a history of devastating natural disasters, such as tropical storms, floods, and droughts, the severity of which is projected to increase with climate change [22,23,24]. The geographical proximity of the Caribbean islands, coupled with their relative proximity to South, Central, and North America, may facilitate the introduction and spread of virus-infected materials and insect vectors [25]. One notable example is the dissemination of tomato begomoviruses from Venezuela through the islands of the Lesser Antilles, up to those of the Greater Antilles, and eventually to Central America and Florida [26]. The movement of the viruses was attributed to the spread of the whitefly vector, *Bemisia tabaci*, during storms and hurricanes, as well as its inadvertent introduction through trade of infected plant material or other material infested with the vector [26].

**Figure 1:**
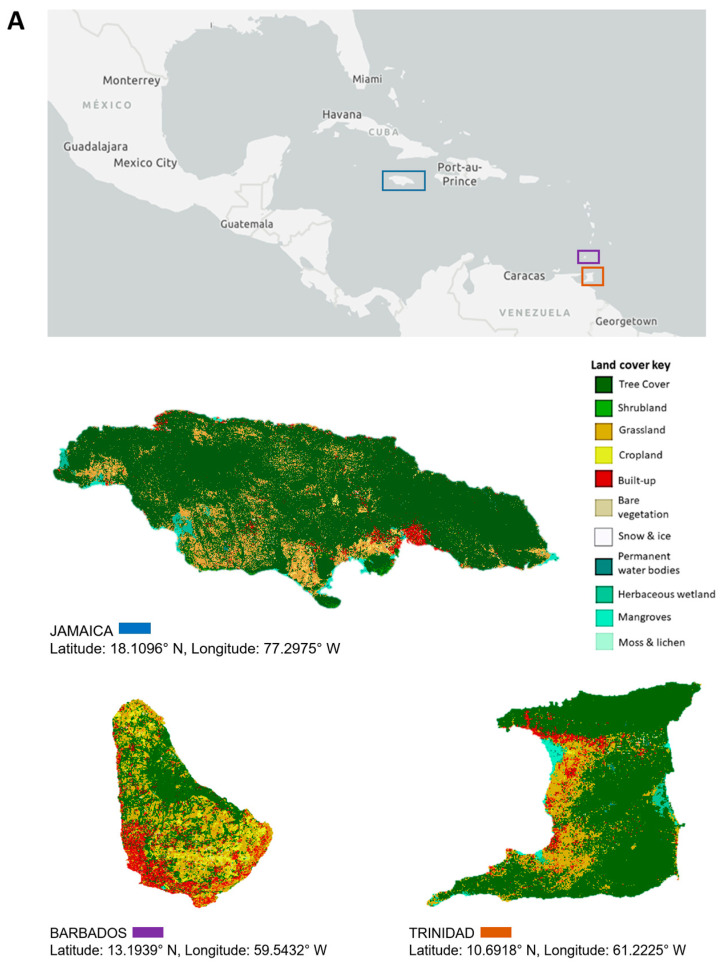

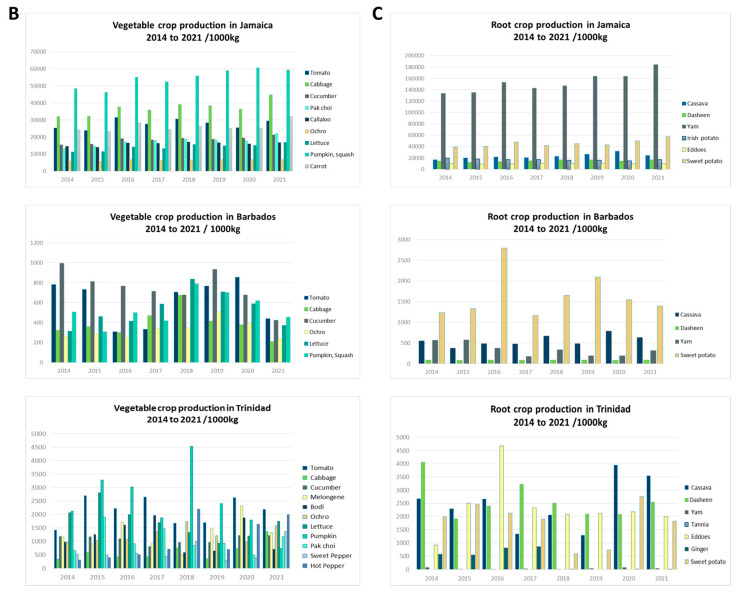
Land use and major crop production between 2014 and 2021 in Jamaica, Barbados, and Trinidad. (**A**) Land use/land cover for Jamaica, Barbados, and Trinidad sourced from the European Space Agency World Cover 2020 dataset. Maps were created in ArGis Online. Major vegetable (**B**) and root (**C**) crop production in Jamaica, Barbados, and Trinidad between 2014 and 2021. Data courtesy of the Ministry of Agriculture, Fisheries and Mining of Jamaica, the Ministry of Agriculture and Food Security of Barbados, and Central Statistical Office (CSO) of Trinidad and Tobago, 2022.

This review focuses on viruses infecting economically important fruit and vegetable crops on three main Caribbean islands, namely Jamaica, Barbados, and Trinidad and Tobago. The three islands are situated at opposite ends of the Caribbean archipelago. Jamaica, part of the Greater Antilles, is positioned south of Cuba and west of Hispaniola [20]. Barbados is the most easterly island in the Lesser Antilles, and Trinidad and Tobago is located at the southernmost end near Venezuela [20]. Jamaica’s total land area covers 1,083,000 ha, Barbados, 43,176 ha, and Trinidad and Tobago, 512,000 ha, with respectively, 39%, 23%, and 11% of their total land mass under agricultural production (Figure 1). Cultivation primarily consists of crops from the families Cucurbitaceae, Solanaceae, and Brassicaceae, as well as root crops belonging to Euphorbiaceae, Convolvulaceae, and Dioscoreaceae (Figure 1). The agriculture industry contributes between 3% and 16% of the total employment in these countries, compared to 7 to 32% in countries of South America and 2% in North America [27,28]. Contemporary agriculture in the Caribbean has become more diversified even though, historically, the countries largely produced similar agricultural commodities, such as sugarcane and bananas, for export [29], and root and tuber crops for local consumption. One of the reasons for this diversification is the reduction of preferences in the international markets for traditional agricultural exports from the Caribbean [29,30]. Jamaica, for example, has diversified its export offerings to include nontraditional crops such as root and tuber crops—yams and sweet potatoes, dasheen, and coco (eddoes). Additionally, it has expanded into fruit trees such as papaya and cucurbits like pumpkin. Local fruit and vegetable production of watermelon, cucumbers, and peppers for domestic use has increased, as well as the cultivation of root and tuber crops such as cassava, Irish potato, and carrots (Figure 1). Table 1 summarizes reports of virus infections in 18 crops grown on the three islands. Some viruses have long been established in the region and are linked to disease epidemics of varying economic significance. Over time, these viruses have become endemic; re-emerge periodically or have the potential to cause future outbreaks (e.g., papaya ringspot virus, citrus tristeza virus, cacao mild mosaic virus, and cacao yellow vein-banding virus). Other viruses are more recent introductions that present ongoing threats to crop production (e.g., begomoviruses, including sweepoviruses) or are only now being recognized and studied (e.g., zucchini yellow mosaic virus and cucurbit yellow stunting disorder virus). This review examines some of the viruses referenced within each of the three groups. These viruses pose major threats due to their prevalence and or the economic losses that they have caused (or are likely to cause) on the three islands and the wider Caribbean region. We present their chronological prevalence to emphasize the agricultural challenges in the region, highlight their impacts, and demonstrate the progression in research. By identifying possible drivers of their emergence, we underscore the importance of early detection, phytosanitary measures, the use of DNA diagnostic technologies, and integrated disease-management approaches for addressing these threats regionally. 

## 2. Endemic and Potential Re-Emerging Threats

### 2.1. Papaya Ringspot Potyvirus

Papaya ringspot virus (PRSV), a single-stranded translational-sense RNA virus, is classified as a member of the genus *Potyvirus* and family *Potyviridae* [70,71]. Two serologically indistinguishable biotypes of PRSV are described [72,73]. Type P is pathogenic to papaya, whereas type W (previously designated as watermelon mosaic virus 1) affects cucurbits. Besides Caricaceae, other hosts of PRSV type P belong to the families Chenopodiacae and Curcubitaceae [45,74,75,76]. However, a broader host range than previously recorded is suggested by the detection of the virus in *Robinia pseudoacacia* L., a tree species in the family Fabaceae [77]. PRSV is spread by several species of aphids [Hemiptera: Aphidadae) in a non-persistent manner [73], and reports indicate a very low rate of seed transmission [77].

According to Jensen [44], symptoms characteristic of PRSV were described on papaya growing in Jamaica as early as 1929. Affected trees were stunted and produced little or no fruit or fruits that were disfigured with sunken ringspot patterns. It was surmised that the virus was the main cause of the absence of papaya cultivation on a large scale on the island. Nonetheless, in the mid-1980s, papaya was promoted in the agricultural sector as a viable alternative to traditional export crops, leading to increased plantings across Jamaica [78]. Shortly thereafter, the first PRSV epidemic was reported in two regions of the island [78]. Government-mandated eradication efforts, which included a restriction on the movement of papaya seedlings and the destruction of all infected plants, temporarily contained the infection; however, blemished fruits destined for export were observed by plant quarantine officers in 1994 [78]. Subsequent analyses of coat protein gene sequences of PRSV isolates from the two outbreaks revealed sequence divergence of up to 8%, suggesting a different origin for each outbreak [45]. Isolates from the early outbreak in the 1990s (GenBank Accession DQ104823) were closely related to those described in the neighboring islands of Cuba (GenBank Accession AY339581 and AY8415757) and Puerto Rico (GenBank Accession AF196838), as well as isolates in Florida (GenBank Accession AF196839) and Venezuela (GenBank Accession DQ339581, EF189734, and EF189736) [45]. Isolates of the second outbreak in the late 1990s (GenBank Accession DQ104812-DQ104818) showed close homology with isolates from Brazil [45]. PRSV diversity appears to be related to geographical location rather than host range and movement, in papaya as well as cucurbits [79]. Several studies suggest that the virus originated from southern Asia, as higher levels of diversity are found among coat protein coding regions of isolates from India and other Asian isolates [80,81,82,83].

Although PRSV is no longer under government biosecurity controls, it continues to limit papaya production in Jamaica [84], and its management remains challenging. Cross protection was not feasible under greenhouse investigations [85]. Virus coat protein-resistant transgenic papaya varieties have not received local regulatory approval for cultivation [86,87,88]. In the absence of resistant varieties, growing papaya in Jamaica involves a combination of quarantine and cultural practices aimed at reducing sources of infection. These include restricted movement of papaya seedlings, scouting of orchards, and prompt removal of infected trees, intercropping with barriers crops (e.g., sorrel (*Hibiscus sabdariffa*) or corn (*Zea mays*), staggering planting dates of the orchards and spraying with mineral oils or insecticides to control insect vectors [89,90,91]. These measures are only effective in locations where the disease pressure is low. The virus is currently not considered a major pathogen of papaya in Trinidad or Barbados. However, with plans underway to increase papaya production in Barbados, the Ministry of Agriculture and Food Security is proactively anticipating potential challenges. Drawing from the experience in Jamaica, they foresee the need for diligent monitoring of orchards to ensure the health and productivity of the crop.

### 2.2. Citrus Tristeza Closterovirus

Citrus tristeza virus (CTV), a closterovirus and member of the family *Closteroviridae*, has also long been associated with the region and continues to pose a threat to citrus industries [92]. CTV possesses the largest non-segmented single-stranded positive-sense RNA genome [93]. Species of *Citrus* and *Fortunella* are natural hosts of CTV, and, depending on the virus variant and the citrus host scion-rootstock combination, the virus can cause two major diseases, quick decline and stem pitting [94]. Quick decline results in the decline and death of scions grafted on sour orange rootstock (*Citrus aurantium* L.), while stem pitting infections affect sweet orange (*Citrus sinensis*), grapefruit (*Citrus paradisi*), lime (*Citrus aurantifolia*), and other citrus varieties irrespective of the rootstock. Although stem pitting rarely results in scion death, it causes loss of vigor leading to drastic yield reductions [93]. Other variants induce mild or indiscernible symptoms in field trees and generally do not substantially affect yields [93]. Recent evidence supports the hypothesis that the virus existed for centuries in Asia [95] and spread to different countries during the global movement of citrus germplasm [93]. New environmental and climatic conditions presumably facilitated interactions with new host varieties, leading to a pandemic that affected multiple countries [93]. The virus ravaged citrus industries in Argentina and Brazil during the 1930s and 1940s [96]. Stell [37] recorded CTV in Jamaica in 1960 [37], and Hosein [38] documented its presence in Trinidad in 1965. However, the virus was not regarded as a threat to the industries at that time, since only mild variants were detected by bio-indexing. Moreover, the primary insect vector, the brown citrus aphid (*Toxoptera citricida* Kirkaldy), was assumed to be restricted to Guyana (which is located on the mainland of South America, northeast of the Caribbean region) despite anecdotal observations of the aphid in the region from as far back as 1949 [96]. As a result, no action was taken to prepare for CTV epidemics by implementing preventative methods until there was evidence of the northward movement of *T. citricida* through the Caribbean [97]. The aphid was identified in Venezuela in 1976; populations were later reported in Trinidad in 1985, Guadeloupe, Martinique, St. Lucia, Puerto Rico, and the Dominican Republic in 1992, and Cuba and Jamaica in 1993 [97]. Rocha-Peña et al. [97] speculated that the increased acreage of citrus in the region facilitated the establishment of *T. citricida*.

By 1997, quick decline symptoms became evident in Jamaica [39], and within 15 years, both quick decline and stem pitting variants were confirmed to be present across the island [40,41,42]. In phylogenetic analyses of the coat protein gene, CTV variants from Jamaica (GenBank Accession GU983384 to GU983389 and HM160500 to HM160-518) were closely related to those from other regions, indicating similarities based on biological properties rather than geographical origin [41,42]. One-third of the Jamaican isolates clustered with the mild Florida-T30 (GenBank Accession AF260651) and Spain-T385 (GenBank Accession DQ151548 and Y18420) genotypes, sharing 99 to 100% identity [42]. The remaining isolates grouped closely with severe Florida T36-like genotypes (GenBank Accession U16304) or with the grapefruit and orange stem pitting B249 from Venezuela and the New Zealand CTV resistance-breaking genotypes (GenBank Accession FJ525431 and FJ525435). Identities among these groups ranged between 91 and 99% [42]. Possibly, there were single or multiple introductions of infected budwood into the island from a region where CTV is widespread [42].

CTV is presently regarded as a regulated non-quarantine pest; consequently, certification programs are still in effect to avoid dissemination of the virus [98]. The management of the virus disease in Jamaica involves monitoring trees for symptoms of CTV, removing infected trees, and replanting on virus-tolerant rootstocks [98]. The industry has apparently benefited from the release of the parasitoid *Lipolexis oregmae* as part of a biological control program in Florida [99]. In 2007, Hoy et al. [100] found two parasitoids, *Lipolexis oregmae* and *Lysiphlebus testaceipes*, infecting Jamaican brown citrus aphid populations. *L. oregmae* oviposits and develops in all four instars of the brown citrus aphid, leading to mortality and the potential regulation of aphid populations [99].

### 2.3. Cacao Mild Mosaic Badnavirus and Cacao Yellow Vein-Banding Badnavirus

Two badnaviruses, cacao mild mosaic virus (CaMMV) and cacao yellow vein-banding virus (CYVBV), have been detected in Trinidad. The source of these viruses in Trinidad is not known. It is speculated that they may be endemic to the island or introduced from virus-infected cacao originating from its center of diversity, which is the upper Amazon region of northwest South America, or through domestication [101,102].

Badnaviruses (family *Caulimoviridae* and genus *Badnavirus*) are among the most economically important and genetically diverse plant virus groups that cause disease in several crops grown in tropical regions, e.g., banana (*Musa acuminata*), black pepper (*Piper nigrum*), cacao (*Theobroma cacao*), citrus (*Citrus* spp.), sugarcane (*Saccharum officinarum*), taro (*Colocasia esculenta*), and yam (*Dioscorea* spp.) [103]. These viruses are transmitted by mealybugs in a semi-persistent manner [103]. Yield losses resulting from infection with viruses of this group vary between 10% and 90% [103,104]. The viruses are non-enveloped, bacilliform DNA viruses that contain a single molecule of non-covalently closed circular dsDNA [105]. At least 11 badnavirus species have been implicated as causal agents of disease in cacao [106].

Whole genome sequences of two badnaviruses infecting cacao in Trinidad, cacao mild mosaic virus (CaMMV) and cacao yellow vein-banding virus (CYVBV) [36,107], were recently studied. These viruses are purported to be the causal agents of distinct virus-like symptoms detected in Trinidad since the 1940s, when they were provisionally called cacao Trinidad virus Strain A and Strain B, based on their symptomology in cacao [34]. Cacao Trinidad virus Strain A (CaMMV) caused red mottling of leaves, while cacao Trinidad virus Strain B (CYVBV) induced yellow vein-banding of leaves [107]. Both the red mottling and vein-banding virus strains are transmitted by four species of mealybugs (Hemiptera: Pseudococcidae): *Pseudococcus citri* (Risso), *P. brevipes* (Ckll.), *P. comstocki* (Kuw.), and *Ferrisia virgata* (Cockerell) [35,108]. The disease was first thought to be restricted to the northwest region of the island, where cacao trees exhibiting symptoms of red mottling and vein-banding were widespread [34]. Island-wide surveys subsequently confirmed the presence of both strains [38]. A government-mandated eradication program in the 1950s, involving the destruction of all infected trees, successfully contained the infection for 14 years; however, red mottling and vein-clearing symptoms were later discovered in trees at the International Cacao Genebank in 2005 [109]. Molecular identification of CaMMV and CYVBV in Trinidad confirmed the first report of cacao-infecting badnaviruses in the Western Hemisphere [36]. It is expected that CaMMV is also the causal agent of virus-like symptoms in other cacao-growing areas in Colombia, the Dominican Republic [110], and Venezuela [111].

Whole genome sequence comparisons of two badnaviruses infecting cacao in Trinidad, namely CYVBV (GenBank Accession NC_033739) and CaMMV (GenBank Accession NC_033738), indicate that these are new genotypes to the GenBank database. CYVBV from Trinidad had only 2% to 10% sequence coverage in the aligned sequences, but it was 99.45% and 98.63% identical (BLASTn) to six other yellow vein-banding viruses in cacao. The closest match to CaMMV from Trinidad had 99 to 100% sequence coverage and 89.7% to 90.42% identity to only three other CaMMV from Puerto Rico and Brazil, respectively, after which sequence coverage in the alignment dropped to 16.00%, with 96.35% identity that corresponded to the polyprotein gene.

**Figure 2:**
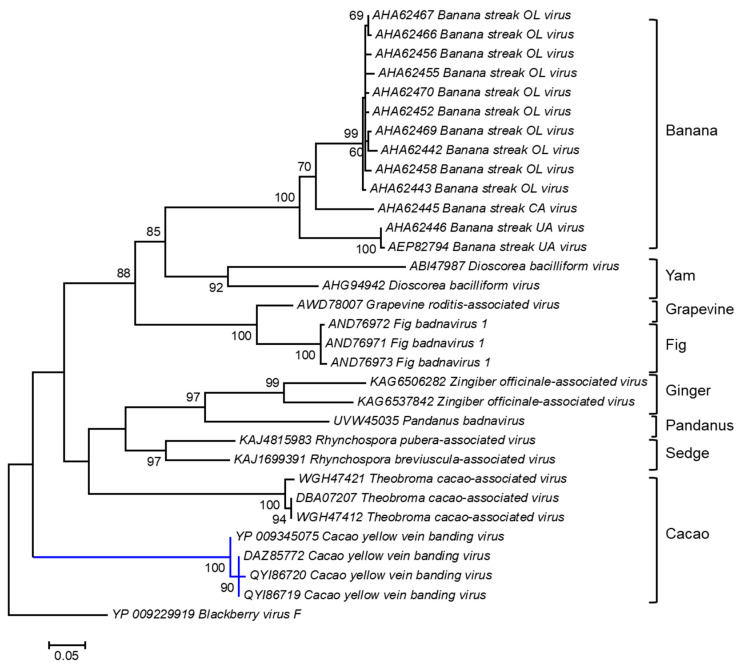
Molecular Phylogenetic analysis of CYVBV and other badnavirus polyprotein sequences using the Maximum Likelihood method. The evolutionary history was inferred by using the Maximum Likelihood method based on the Le_Gascuel_2008 model [114]. The tree with the highest log likelihood (-3934.2996) is shown. The percentages of trees in which the associated taxa clustered together are shown next to the branches. Initial tree(s) for the heuristic search were obtained automatically by applying Neighbor-Joining and BioNJ algorithms to a matrix of pairwise distances estimated using a JTT model and then selecting the topology with superior log likelihood value. A discrete Gamma distribution was used to model evolutionary rate differences among sites (5 categories (+G, parameter = 1.0715)). The tree is drawn to scale, with branch lengths measured in the number of substitutions per site. The analysis involved 32 amino acid sequences. All positions containing gaps and missing data were eliminated. There were a total of 259 positions in the final dataset. Evolutionary analyses were conducted in MEGA6 [115].

A cladogram was inferred based on the amino acid sequences of the polyprotein of badnaviruses infecting different plant host species, including cacao (Figure 2). The polyprotein sequences of three of the four Trinidad CYVBV, highlighted by the blue branch, were nearly identical, with 99.25% and 100% amino acid sequence similarity. YP_009345075 shared 98.13% similarity with the other three Trinidad viruses. The Trinidad viruses were positioned with high bootstrap support (100%) into a discrete cluster consisting of three endogenous badnaviruses infecting cacao (GenBank Accession WGH47421, DBA07207, and WGH47412). Endogenous viral elements (EVEs) are viral sequences (whole or partial) that have become integrated into the host genome; subsequent replication of the host genome fixes these EVEs such that daughter cells now carry the entire complement of the host genome with EVE elements [112]. The integration of functional full-length viral sequences into host genomes can trigger a response to systemic virus infection and affect the expression of specific host genes, e.g., the banana streak virus (*Badnavirus*) in bananas [113].

**Figure 3:**
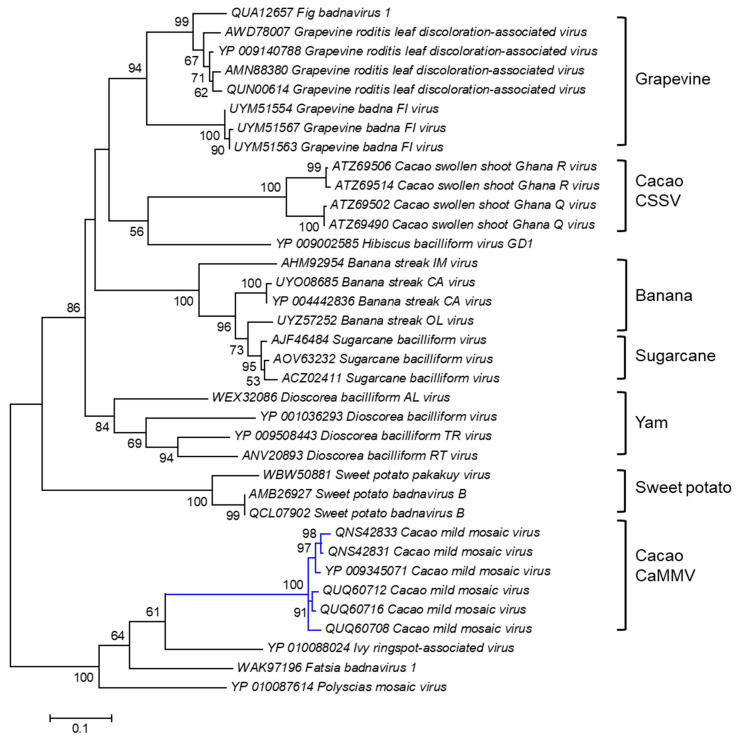
Molecular Phylogenetic analysis of CaMMV and other badnavirus polyprotein sequences by the Maximum Likelihood method. The evolutionary history was inferred by using the Maximum Likelihood method based on the Le_Gascuel_2008 model [114]. The tree with the highest log likelihood (-7360.0517) is shown. The percentages of trees in which the associated taxa clustered together are shown next to the branches. Initial tree(s) for the heuristic search were obtained automatically by applying Neighbor-Joining and BioNJ algorithms to a matrix of pairwise distances estimated using a JTT model and then selecting the topology with superior log likelihood value. The analysis involved 36 amino acid sequences. All positions containing gaps and missing data were eliminated. There were a total of 408 positions in the final dataset. Evolutionary analyses were conducted in MEGA6 [115].

A cladogram was Inferred based on the amino acid sequence of the polyprotein of CaMMV and other badnaviruses (Figure 3). Six polyprotein sequences of the Trinidad viruses, highlighted by the blue branch, were positioned with high bootstrap support (100%) into a discrete cluster. The polyprotein sequences of CaMMV infecting cacao in Trinidad were also distinct from another cacao-infecting badnavirus called cacao swollen shoot virus, CSSV, which causes severe disease in cacao grown in West African countries [116].

The difficulty in diagnosing this disease and managing these viruses in cacao lies in their ability to infect cacao without necessarily causing visible foliar symptoms [117]. Additionally, Ullah et al. [107] confirmed that the titer of CaMMV particles in infected trees is lower in asymptomatic hosts with uneven distribution among the leaves of a single tree, thereby challenging virus DNA extraction for molecular diagnosis. This has practical implications for the exchange of cacao germplasm between breeding centers and botanical gardens [118].

Approaches to the detection of the viruses in Trinidad include a combination of visual inspection of plant material for symptoms of infection and testing using molecular techniques [107]. A novel assay for the detection of CaMMV and CYVBV in Trinidad has been developed. The method is based on the colorimetric Loop-mediated isothermal amplification (LAMP) assay [107]. This method could be employed in virus testing to offer several screening advantages: (i) the method is effective in detecting viruses in non-symptomatic accessions, (ii) this molecular approach is simple and accurate, (iii) the assay is sensitive enough for the detection of viruses at low virus titers, and (iv) the method has the potential for use in field testing [107]. The presence of endogenous badnaviruses poses a new challenge to the development of diagnostic tests and management of the diseases, especially in the context of extensive global exchange and the transfer of cacao germplasm [119].

## 3. Recent and Ongoing Threats

### 3.1. Tomato Yellow Leaf Curl Begomovirus

The begomovirus and member of the *Geminiviridae*, Tomato yellow leaf curl virus (TYLCV), causes severe disease in several Solanaceous crop plants in the Caribbean (Table 1) and elsewhere [120]. TYLCV is a small, circular single-stranded DNA virus of ~2700 nts [121]. The virus genome is associated with α-satellites and β-satellites [121]. Alpha-satellites are about 1.4 kb. They encode a replication-associated protein and are known to reduce TYLCV symptom expression in host plants, as well as β-satellite accumulation. Beta-satellites are 1.3 kb and encode a single protein, βC1, which functions as a suppressor of transcriptional gene silencing [121]. Phylogenetically, two major groups of begomoviruses are distinguished [122,123]: begomoviruses native to the Caribbean and the Americas (New World) and those (Old World) that are wide-spread in Africa, Asia, Europe, and Oceania. Some of the New World viruses and most of the Old World viruses are associated with sub-genomic DNA components [124,125,126]. The emergence of begomoviruses as important pathogens over the past three decades is associated with the increased prevalence of their vector [127,128]. The transmission occurs exclusively by the whitefly vector *Bemisia tabaci* (Gennadius) (Hemiptera: Aleyrodidae) in a persistent-circulative or persistent-propagative manner [129,130]. The cryptic species MEAM1 (formerly known as biotype B) and MED (formerly known as biotype Q) are the most efficient transmitters [3,131]. *B. tabaci* MEAM1 has been identified in the Lesser Antilles, i.e., Dominica, Grenada, Guadeloupe, Martinique, Montserrat, St Kitts and Nevis, Trinidad, and St. Vincent [132]. The polyphagous MEAM1 is reported to have replaced the less polyphagous local A biotype [26]. The transovarial transmission of TYLCV occurs with both the MEAM1 and MED biotypes [133]. Guo et al. [134] detected TYLCV virions in *B. tabaci* eggs; however, the virus was not detected in the nymphal stage, which indicates the inability of the adult stage to transmit the virus. Kil et al. [135] demonstrated the possibility of seed transmission. The virus has a diverse host range and has been found in 49 species belonging to 16 families [136].

TYLCV was reported in Jamaica in 1994 [55,137], in Barbados in 1998, and in Trinidad in 2014 [138]. Mabvakure et al. [131] suggested that TYLCV may have been introduced into the United States via the Caribbean in the early 1990s, which coincides with serious TYLCV outbreaks in the Dominican Republic [139]. TYLCV in the Dominican Republic is closely related to TYLCV-IL from the Eastern Mediterranean [140]. The virus presumably moved from the Dominican Republic into Cuba, to Jamaica, and then to the United States [139]. Mabvakure et al. [131] described three possible introduction events into the Caribbean from Western Mediterranean, Eastern Mediterranean, and East Asia based on phylogeographic analyses, whereas two introduction events (from the Mediterranean and Asia) were identified by Lefeuvre et al. [140]. Their data were based on the approximate dates when recombination events may have occurred and identification of the geographical origins of the ancestral recombinants. The introduction of TYLCV-Israel into the Caribbean from East Asia occurred between 2006 and 2011, and TYLCV-Mld, a mild strain of TYLCV, may have been introduced into the Caribbean from the Western Mediterranean between 1990 and 2009, based on phylogeographic inferences [131].

Evidence for the contribution of the Caribbean in the distribution of TYLCV to the United States lies in strongly supported temporal and phylogeographic relatedness of the United States isolates and isolates from Cuba, Dominican Republic, Guatemala, and Puerto Rico, which are most closely related to TYLCV isolates from Jordan, Israel (TYLCV-IL) [140]. The trans-global distribution of TYLCV can be attributed to the anthropogenic movement of virus-infected plant material, including seeds or infected fruit [141] and/or the inadvertent introduction of TYLCV-viruliferous whiteflies [142,143]. Based on the foregoing, it was purported that virus introductions into the Caribbean make this region a hotspot for virus diversification [144].

**Figure 4:**
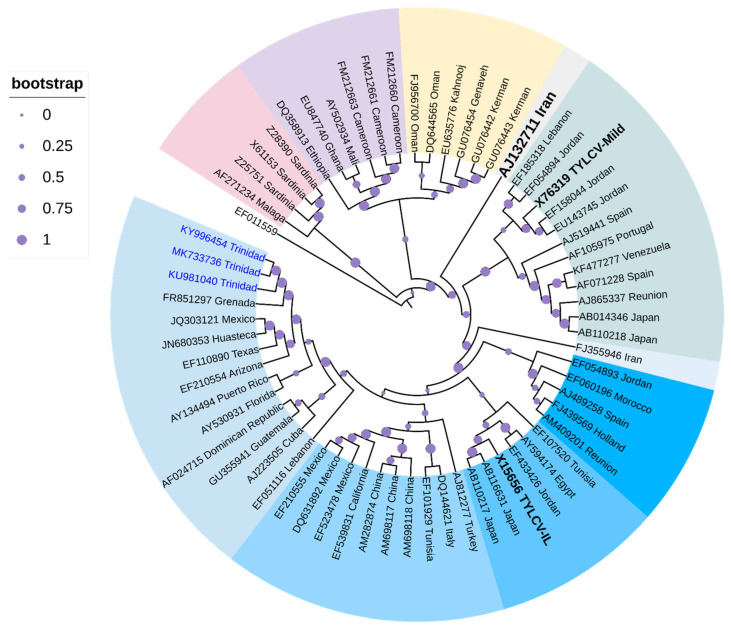
Cladogram for TYLCV based on Maximum Likelihood. The evolutionary history was inferred by using the Maximum Likelihood method based on the General Time Reversible model [145]. The tree with the highest log likelihood (-20,238.5922) is shown. The analysis involved 66 nucleotide sequences. All positions containing gaps and missing data were eliminated. Evolutionary analyses were conducted in MEGA6 [114]. The cladogram was re-drawn in iTol version 6.

Cluster analysis (Figure 4) positioned three TYLCV strains from Trinidad (highlighted in blue-labeled taxa) within the TYLCV-IL clade, along with other strains from the Caribbean Basin, i.e., Grenada, and Guadeloupe, two strains from Mexico, and strains from Arizona and Texas with high support (94%). Strains from Cuba, Dominican Republic, and Guatemala, together with strains from Puerto Rico and Florida, seemed to have been positioned into two sub-clades of this Caribbean TYLCV-IL cluster but with bootstrap support <70%. One strain from Venezuela was positioned in the TYLCV-Mld clade that was closely related to a strain from Spain (GenBank Accession AF071228) with high bootstrap support (93%). There was clear separation of TYLCV-IL from TYLCV-Mld and TYLCV-IR (in black bold-labeled taxa).

In the Caribbean, the primary crop affected by TYLCV is tomato. Infected plants show severe stunting, leaf curling, and yellowing symptoms, which cause critical production losses. The estimated losses range from 30 to 100% across the Caribbean islands, Mexico, Central America, and Venezuela; yield loss is the main impetus for the establishment of robust quarantine measures [121]. The successful management of TYLCV is dependent on integrated disease-management practices before, during, and after the growing season, in addition to the use of TYLCV-tolerant tomato cultivars [146]. Wild tomatoes with resistant genes Ty-1/3, Ty-2, Ty-4, ty-5, and Ty-6 [121,146,147,148,149] were used to develop commercially available TYLCV-tolerant cultivars. Four of these cultivars are currently grown in Jamaica, cvs. Summer Star, Striker, AMSA 425, and Tropical Glory (https://jis.gov.jm/bodles-produces-new-disease-resistant-tomatoes/, accessed on 15 January 2024). These cultivars offer the advantage of producing more marketable tomatoes while developing only mild symptoms of TYLCV infection. It is therefore crucial to balance the use of these cultivars with other disease-management strategies and continuous monitoring of TYLCV [150,151].

In Trinidad, seeds of tomato cultivars with varying levels of tolerance to TYLCV (e.g., TYLCV +: cvs. F1 TX100, TYLCV ++: cvs. F1 IT102, Sherry F1/CH7, F1 TX40, F1 TX45, F1 TX68; TYLCV +++: cvs. F1 TX54, F1 TX62, F1 TX65) are currently available commercially for farmers (http://mafascaribbean.com/home/tomato/, accessed on 22 November 2023). Seeds of tomato cultivars with zero-listed TYLCV tolerance and resistance to a number of important fungal diseases (e.g., *Fusarium* sp., *Verticillium* sp., *Alternaria* sp., *Stemphylium* sp.) and other viruses (e.g., tobacco mosaic virus (TMV) and tomato spotted wilt virus (TSWV) are also available. Farmers prefer to grow ‘dual purpose cultivars’, e.g., ‘Hybrid 61’ and ‘Mungal’, even though they are not TYLCV-tolerant mainly because these varieties produce many fruits per cluster, are less susceptible to physical injury, exhibit more uniform ripening, and have a longer shelf-life; these preferred traits may be a compromise for an estimated postharvest yield loss of 27% due to TYLCV infection [152].

### 3.2. Sweet Potato Feathery Mottle Potyvirus and Sweet Potato Chlorotic Stunt Closterovirus

Synergistic interactions between sweet potato feathery mottle virus (SPFMV) and sweet potato chlorotic stunt virus (SPCSV) result in sweet potato virus disease (SPVD) in sweet potato (*Ipomoea batatas*) [153,154]. Jones [155] suggested that both viruses likely co-evolved with cultivated sweet potatoes in one of two centers of domestication (Central or South America) and were distributed globally through infected tuberous roots. East Africa is also a secondary hotspot for virus diversity in sweet potatoes based on evidence of widespread infection in weeds and alternative host species [156,157,158].

SPFMV is a positive-sense single-stranded RNA potyvirus of the family *Potyviridae* [159,160]. SPCSV, a closterovirus with a bipartite genome, possesses the second largest single-stranded positive RNA genome [161,162,163]. SPCSV is transmitted by whiteflies in a semi-persistent manner, whereas the transmission of SPFMV occurs through various species of aphids in a non-persistent manner [153,154]. On their own, the viruses induce mild symptoms in sweet potatoes that are often confused with nutrient deficiencies [153,161]. The primary impact on the crop occurs in co-infections. Plants infected with SPFMV and SPCSV exhibit leaf reduction, deformation, and stunting symptoms, which contribute to decreased usable yield and severe economic losses [153]. SPCSV is also capable of mediating severe synergistic viral diseases with other sweet potato-infecting viruses, such as sweet potato mild mottle virus (SPMMV; genus *Ipomovirus*) [164], sweet potato virus G (SPVG; genus *Potyvirus*) [165], and cucumber mosaic virus (CMV; genus *Cucumovirus*) [166]. Synergism between SPCSV and sweepoviruses has also been reported [167].

**Figure 5:**
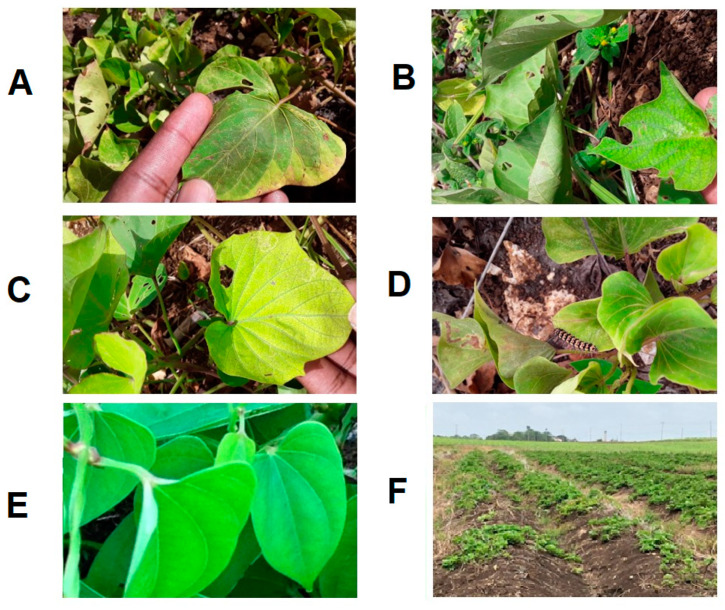
Foliar symptoms on sweet potato in Barbados. (**A**): Vein clearing, insect damage, and chlorosis; (**B**): Leaf curling, crinkling, and deformation; (**C**): Chlorosis and vein clearing; (**D**): Vein clearing, leaf curling, and insect damage; (**E**): Disease-free, healthy leaves; and (**F**): Yield decline in Barbados.

James et al. [64] first reported the presence of SPFMV and SPCSV co-infections in diseased fields in Barbados. In that study, NCM-ELISA testing showed the presence of single infections of SPMFV or sweet potato virus 2 (SPV2) [65], but mixed infections with both viruses were frequent [65]. These plants displayed stunting, leaf distortion, and pale mosaic or vein clearing. Some cultivars also displayed purple mottling on lower leaves (Figure 5). In addition, triple virus infections comprising mixtures of SPVG, SPV2, and SPCSV caused severe stunting in infected plants. However, those samples that tested positive for SPFMV alone were asymptomatic or showed vein clearing [65].

Since this initial survey of the southern and central regions of the island in 2001, virus disease symptoms have been reported across the entire island, affecting both commercial operations and small holdings in all three agroecological zones [63]. SPFMV was the only virus detected by RT-PCR. When these amplified fragments were sequenced, SPFMV was confirmed. BLASTn analysis of the sequences revealed 96% nt identity with a SPFMV isolate from Kenya (GenBank Accession AY523547) and 100% identity with the polyprotein gene of an isolate from Uganda (GenBank Accession FJ795751). Analysis of high-throughput sequencing (HTS) data suggested the absence of the viruses involved in SPVD and, instead, possibly new, severe single virus infections or emerging virus associations, largely composed of sweepoviruses, badnaviruses, potyviruses, and mastreviruses, previously unreported in sweet potato in the Caribbean [63]. Among these were several begomoviruses representing SPLCV and IYVV, which grouped into two separate clades sharing 85–98% homology with isolates from Spain (GenBank Accession EU839577 and EU839576.2), South Korea (GenBank Accession HM7546337), Puerto Rico (GenBank Accession DQ 644562), North America (GenBank Accession AF326775), and Brazil (GenBank Accession no. HQ393460) [63].

Prior to 2004, virus diseases of sweet potato in Jamaica were largely overlooked, with primary attention directed towards managing insect pests. However, a report published in 2004, which identified an undetermined geminivirus [62], prompted further investigations into the viruses harbored in the crop. Subsequent surveys of the major sweet potato growing regions, where apparently healthy vines were observed, revealed the presence (2–78%) of potyviruses, carlaviruses, criniviruses, cucumoviruses, and a caulimo-like virus in NCM-ELISA tests [63]. In follow-up surveys conducted five years later (2011) in the same fields, the majority of the vines (95%) exhibited vein-clearing symptoms and leaf deformation [60]. Infections with SPFMV, SPCSV, sweet potato chlorotic fleck virus (SPCFV), and SPMMV (<5%) were confirmed, and sweet potato collusive virus (SPCV) and CMV were most frequently detected, at 23% and 42%, respectively. Mixed infections with CMV, SPCV, and sweet potato mild speckling virus (SPMSV) or CMV, SPCV, SPMSV, and sweet potato latent virus (SPLV) were also found in a third of the samples tested [60]. The findings of both surveys suggested the absence of the SPVD complex in Jamaica. Like the Alleyne et al. study [63] in Barbados, analysis of HTS data revealed species of potyviruses, sweepoviruses, badnaviruses, and mastreviruses [61]. However, sweepoviruses proved more prevalent in the sweet potato virome in Jamaica, and the solendovirus, sweet potato vein clearing virus (SPVCV), was detected for the first time in the region [61].

Disease management has focused on the use of clean planting material derived from tissue culture. In 2021, the Ministry of Agriculture and Food Security and the University of the West Indies campus in Barbados, in collaboration with the Food and Agriculture Organization of the United Nations (FAO), launched a project aimed at training farmers and establishing plant nurseries for producing clean planting material through tissue culture [168]. Ongoing farmer training, both formal and informal, includes activities such as identifying disease symptoms, managing weeds, and raising awareness about wild *Ipomoea* species that can host sweet potato viruses. Breeding for resistance, while possible, is currently not employed, even though the presence of resistance genes in locally bred cultures has been reported [169].

## 4. Newly Recognized Threats

### 4.1. Zucchini Yellow Mosaic Potyvirus

Potyviruses (Family *Potyviridae*, Genus *Potyvirus*) are the predominant plant virus species in Trinidad, infecting the majority of economically important crop species grown (Table 1). They are transmitted by aphids (Hemiptera: Aphididae) [170] and represent the largest group of plant-infecting viruses. The RNA genome of potyviruses affords these viruses the highest rates of mutation and evolution, as well as the highest degree of genetic diversity, compared to dsDNA viruses [171]. Regions of genome hyper-variability confer adaptive flexibility that may contribute to host adaptation, host-dependent pathogenicity, vector transmissibility, and high relative replication rates/viral load in different host plants [172].

**Figure 6:**
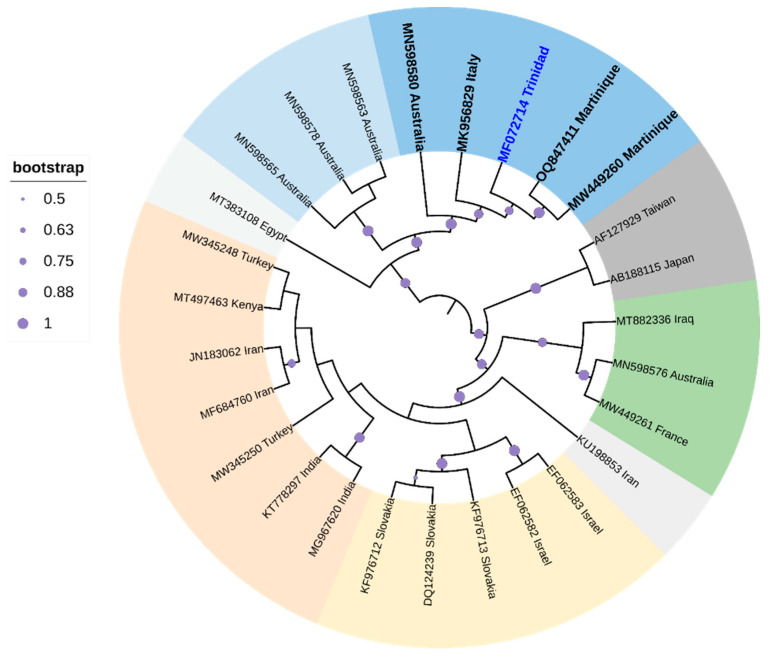
Cladogram for ZYMV based on Maximum Likelihood. The tree with the highest log likelihood (-31,449.6166) is shown. The analysis involved 27 nucleotide sequences. All positions containing gaps and missing data were eliminated. There were a total of 9451 positions in the final dataset. Evolutionary analyses were conducted in MEGA6 [115]. The cladogram was re-drawn in iTol version 6.

One species, zucchini yellow mosaic virus (ZYMV), infects various cucurbits in Trinidad [173]. Symptoms typically include foliar deformation and mosaics, along with fruit deformation [174]. The single-stranded translational-sense RNA virus [174 is transmitted non-persistently by *A. gossypii* (Glover), *A. citricola* (Patch), *Macrosiphum euphorbiae* (Thomas), and *Myzus persicae* (Sulzer) and seeds [175]. Cluster analysis, based on whole genome sequence comparisons, positioned the ZYMV strain from Trinidad (GenBank Accession MF072714; highlighted in blue-labeled taxa in Figure 6 closest to two ZYMV strains from Martinique. The branch length of the ZYMV strain from Trinidad suggested that a large amount of genetic change accumulated in the genome over evolutionary time with strong support (84%) for divergence from the closest whole genome relatives from Martinique (GenBank Accession MW449260 and OQ847411). Sequences from Trinidad, Martinique, Australia, and Italy clustered in a strongly supported clade (99%). Two other strains appeared to have accumulated more genetic change throughout evolutionary time with high support (99%), and these strains were isolated from Taiwan (GenBank Accession AF127929) and Japan (GenBank Accession AB188115). Chinnadurai et al. [173] suggested that this ZYMV from Trinidad should be considered a new genotype.

Management of potyviruses in Trinidad requires the use of virus-free planting material, virus-resistant cultivars, the implementation of physical barriers to block movement of the vector, and the rapid disposal of inoculum sources, e.g., weed species, away from planting sites [176,177]. The use of barrier crops, oil spraying, straw and reflective mulching, and intercropping have all been reported as effective in controlling the vector and, subsequently, in controlling virus populations for a number of viruses in different cropping systems [178,179,180,181].

There is limited information on the virus on other islands of the Caribbean. Thomas [67] detected the virus in pumpkin fields in Jamaica, either as single infections or in combination with the potyvirus, watermelon mosaic virus (WMV). Mixed infections with ZYMV, WMV, and squash mosaic virus (SqMV) were also detected [67]. Co-infections with ZYMV and SqMV, have been reported in St. Vincent, Guadeloupe, St. Lucia, and Dominica [68]. Desbiez et al. [182] attributed the trade of cucurbit plant materials and possibly seeds to the introduction of ZYMV in Martinique and Guadeloupe. Multiple introductions or interactions between different variants presumably account for ZYMV diversity in Guadeloupe and Martinique, respectively [182]. Lecoq et al. [183] describe ZYMV as one of the most damaging emerging viruses, and its recent presence in the Caribbean has been confirmed.

### 4.2. Cucurbit Yellow Stunting Disorder Crinivirus

Cucurbit yellow stunting disorder virus (CYSDV) is another virus recently detected in the Caribbean. The virus consists of a bipartite, positive single-stranded RNA genome; RNA1 encodes genes involved in virus replication, while RNA2 encodes genes for encapsidation, movement, and transmission [184]. CYSDV belongs to the genus *Crinivirus* and family *Closteroviridae* [184]. First identified in the United Arab Emirates in the 1982 [185], CYSDV has since spread to a number of countries in the Mediterranean region and North America, causing severe economic damage and losses [186]. The increased incidence of CYSDV in the last 20 years has been attributed to increased whitefly (*B. tabaci*) populations, increased movement of both the vector and the host, and increased secondary or reservoir hosts [187]. Initially believed to be limited to cucurbits (cucumber, pumpkin, watermelon, melon), a number of other hosts have been identified: snap bean (*Phaseolus vulgaris*), alfalfa (*Medicago sativa*), and lettuce (*Lactuca sativa*). *Physalis wrightii* (ground cherry) has been identified as a potential weed host [187]. Other weed hosts include pigweed (*Amaranthus* sp.), london rocket (*Sisymbrium irio*), nightshade (*Solanum nigrum*), and alkali mallow (*Sida hederacea*) [188].

In 2018, yellowing symptoms, typical of crinivirus infection, were observed on cucurbit crops, cantaloupe (*Cucumis melo*), watermelon (*Citrullus lanatus*), and cucumber (*Cucumis sativus*) on commercial farms located in the principal vegetable-producing region of Jamaica [69]. Infection with CYSDV was confirmed by RT-PCR [69], and an isolate from cantaloupe (GenBank Accession OR399555) was found to share identical RNA1 sequences with isolates from Arizona and Georgia, United States (GenBank Accession EF547827 and MW147553] and >99% similarity to isolates from Southern Europe (GenBank Accession AY242077 and OL584359) and the Middle East (GenBank Accession OR589766).

Apart from the initial report of CYSDV in Jamaica [69], little is known about the prevalence, molecular characteristics, and diversity of CYSDV in the Caribbean. There is need to initiate surveys for comprehensive monitoring of the virus as well as other criniviruses in the region. These viruses are not only known to induce devastating diseases when present as single infections; they can also remain asymptomatic and only lead to disease when involved in mixed infections with other viruses [189]. One notable example is the SPVD described earlier, which results from co-infection of the crinivirus, SPCSV, and the potyvirus, SPFMV. Synergistic interactions of sweet potato begomoviruses with criniviruses have also been documented [167]. Additionally, the vector [190] has been identified on several islands in the Lesser Antilles [132].

## 5. Challenges and Perspectives

In the Caribbean, farmers cultivate crops that serve as hosts to several viruses ranked as economically important globally [12]. Re-emerging viruses, new viruses, and new variants are a constant challenge [191]. With increasing interest in cultivating non-traditional crops, it is inevitable that disease problems will continue to arise. While this review focused on a subset of viruses in four families, some of which exhibit unique genomic signatures, a range of viruses from more than 10 families affect agricultural crops of economic importance to the region (Table 1), with many documented based solely on symptoms induced in crops without laboratory confirmation (Table 2). Caribbean islands face challenges in safeguarding their food security as well as biodiversity. The islands are regarded as a biodiversity hotspot [192], encompassing not only plant and animal diversity but also sustaining microbial diversity. Implementation of the triad of prevention, detection, and control is therefore critical for crop protection and disease management.

### 5.1. Quarantine and Biosecurity Measures

Phytosanitary measures are important for protecting agricultural systems by reducing the spread of pathogens among the Caribbean islands and facilitating international trade in plant products [194]. Each island within the Caribbean has in place a Plant Quarantine Unit acting as their National Plant Protection Organization (NPPO) responsible for monitoring and inspecting all planting material, plants, and their products entering and leaving their countries. Some islands, such as Jamaica and Trinidad, have larger facilities/divisions and employ multipronged approaches, including inspections, surveys, testing and field monitoring, and training. Trinidad and Barbados adhere to stricter protocols that include the exclusion of certain goods and travel. Distinct lists for quarantine purposes follow various International Standards for Phytosanitary Measures (ISPMs). These lists consist of regulated quarantine pest lists, regulated non-quarantine pest lists, and pest lists specific to the particular island that are derived from disease surveys and pest analysis studies [195]. Diseases of major economic importance are also discussed on a regional level every few years through the United States Department of Agriculture [196]. In 2021, the 11th European Development Fund (EDF) ISPMs Project was launched to increase compliance with many SPS Measures across the islands.

Although countries in the region have established quarantine systems, their effectiveness is often constrained by limited financial, human, and infrastructural resources. Collaborative efforts at regional and international levels [197], involving researchers, extension personnel, plant biosecurity organizations, funding bodies, and policy makers, are necessary and would benefit the region [155,198].

### 5.2. Early Detection and Diagnosis

Sensitive yet rapid detection methods specific to target virus(es) are pivotal to both pre-quarantine and post-quarantine measures, as well as for evaluating disease-management protocols [19]. Since the 1990s, serological-based tests and PCR-based methods have become more widely used in the region, expanding the understanding of viruses infecting agricultural crops (Table 1). As described in preceding sections, these methods effectively assessed the prevalence and distribution of CTV variants throughout Jamaica and identified badnaviruses affecting cacao in Trinidad. Similar applications are crucial for confirming the presence and distribution of viruses in the region, particularly those identified solely through symptomatology (Table 2). The confirmation of species from more than 10 genera is required, as well as other viruses and variants that are likely to emerge.

However, diagnosis is complicated by the frequent presence of multiple viruses in plants (Table 1). Many plant viruses are generalists and infect different crop plants, including wild plants [199,200], and many vectors are polyphagous and capable of transmitting multiple viruses to the same plant [201]. Case in point, sweet potato crops in Barbados and Jamaica host more than nine species belonging to four genera (Table 1). Another complicating factor is the presence of asymptomatic plants. Virus infection may not always evoke symptom expression in the host plant; this may be due to neutral or beneficial interactions with the host, latent infections, host tolerance, or environmental factors [202]. This presents a challenge for visual detection in the field but also for inspectors of fresh produce at the border. Methodologies that enable quick detection, especially of novel or emerging pathogens, and the accurate diagnosis of multiple viruses are essential. While HTS and real-time PCR are probably still too expensive for routine diagnosis in the region, group-specific degenerate primers in PCR and multiplex PCRs could facilitate broad spectrum detection [203,204]. Rapid, simple, and inexpensive tests such as LAMP coupled with lateral flow devices can also be used directly in the field [205,206].

**Figure 7:**
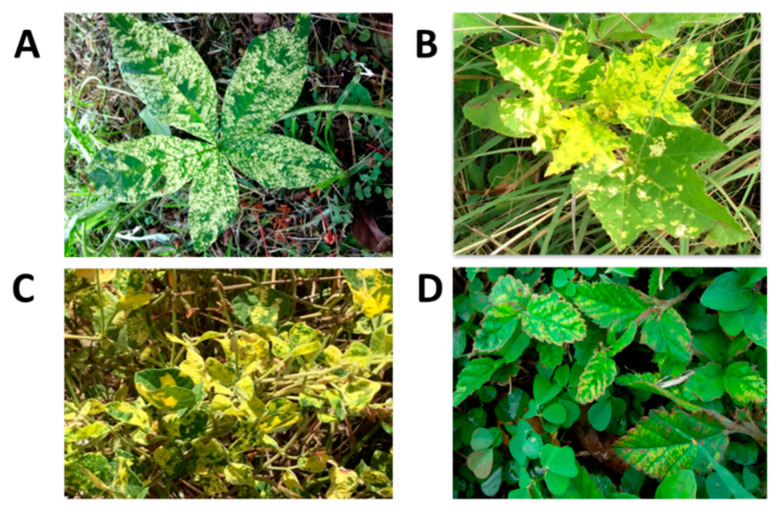
Foliar symptoms of yellow mosaic due to begomovirus infection in (**A**): *Merremia aegyptia*, (**B**): *Malachra alceifolia*, (**C**): *Rhynchosia minima*, and (**D**): *Sida acuta* in Trinidad. Begomovirus infection was confirmed using immuno-capture PCR (IC-PCR).

Nucleic acid-based tests have facilitated the establishment of origins of outbreaks (e.g., CTV in Jamaica), sources of virus introductions (e.g., TYLCV), and analysis of virus populations (e.g., TYLCV). Such knowledge informs decisions on whether to adjust quarantine measures associated with international trade and germplasm exchange, as well as guide choices related to control measures [207]. Despite some progress in understanding the diversity of viruses in crops, wild plant communities, including weeds, have been largely overlooked. In the Caribbean, non-cultivated plants belonging to a wide range of families—Asteraceae, Capparaceae, Convolvulaceae, Euphorbiaceae, Fabaceae, Malvaceae, Nyctaginaceae, and Solanaceae—can be found in or around cultivated areas, exhibiting symptoms of virus disease [173]. Weed species are potential reservoirs for viruses and contribute to the distribution, ecology, and emergence of viruses [208,209]. This is especially true for begomoviruses in the region; weeds serve as reservoirs for these viruses, e.g., [210,211], and these alternate hosts are regarded as integral to begomovirus evolution. Cabbage leaf curl virus (CabLCJV]), for example, is possibly a result of recombination following mixed infections with different bipartite begomoviruses in a common host [53,212]. The virus (CabLCV), originally described in Florida [213], was isolated in Jamaica (CabLCV-JM). CabLCJV was likely generated from a recombination event between CabLCV-[JM] and the weed virus macroptilium golden mosaic virus-Jamaica Strain 1 MaGMV [JM1] [53]. CabLCJV, together with CabLCV-[JM] is associated with severe leaf curling in cabbage that results in the malformation of the ‘cabbage head’ [53]. In Trinidad, potato yellow mosaic Trinidad virus-[Trinidad and Tobago] (PYMTV-TT) was found in five weed hosts [54]. Merremia mosaic Trinidad virus appears to be a cryptic species with a DNA-A genome that contains recombination footprints of the okra yellow mosaic virus from Mexico (GenBank Accession EF591629), rhynchosia rugose golden mosaic virus from Mexico (GenBank Accession NC_038805), and sida yellow mottle virus from Cuba (GenBank Accession NC_016082). Rampersad and Umaharan [214] reported on *Rhynchosia minima* and *Sida* sp., as weed hosts to two begomoviruses in Trinidad. Based on core coat protein alignments, rhynchosia mottle virus from Venezuela (GenBank Accession OK044488) was the most similar to the begomovirus infecting *R. minima* in Trinidad. *Sida acuta* and *S. rhombifolia* were weed hosts to two begomoviruses that were most similar to sida golden mosaic virus from the United States (GenBank Accession MK089808) but showed genetic evidence of PYMTV-TT based on the rep/cp region of the genome. Typical foliar symptoms induced in these weed hosts as a result of begomovirus infection are illustrated in Figure 7. Viruses in weed species growing in Jamaican sweet potato fields or in their immediate proximity could potentially serve as reservoirs for severe synergistic infections in the sweet potato crop [66]. Eight viruses have been detected in perennial plants of the family Convolvulaceae, namely *Merremia umbellata*, *Merremia dissecta,* and an unknown species of *Ipomoea*. SPFMV, sweet potato C6 virus (C-6), CMV, SPCV, and SPMSV were commonly detected; one Convolvulaceae weed sample was seropositive for SPCSV. SPMMV was detected in Solonaceae and Amaranthaceae, namely *Solanum americanum* and *Amaranthus* spp., respectively [66].

The converse is also true; increasing evidence suggests that crops may act as reservoirs for the emergence of viruses in wild plants [208,209]. More surveillance using molecular diagnostic tools is required throughout the region to advance the understanding of viruses circulating in both non-cultivated and cultivated plants. Assessing virus diversity, monitoring evolutionary dynamics, and identifying epidemic-associated factors of economically important viruses could aid in forecasting the emergence of new variants and in mitigating the severity of disease outbreaks [215]. Continued regional and international cooperation will be indispensable [216].

### 5.3. Integrated Disease Management (IDM)

There are a growing number of ecologically sustainable strategies to better manage diseases that are compatible with local cropping practices [217]. Integrated strategies that combine chemical, cultural, and host-resistance approaches (IDM) are the mainstay of disease control [216,217,218]. Additionally, the importance of shifting from extension services focused on the transfer of technologies to improved extension services involving farmer participation and experimentation within farming communities is recognized [219]. Work in Jamaica’s neighbor, the Dominican Republic, involving the management TYLCV with a combination of host-resistance, cultural, and chemical methods, including a three-month whitefly host-free period, is a notable IDM example [218]. Although the planting of resistant cultivars is an important component of IDM, access may be limited in the region. Where available, local seed suppliers do promote new disease-resistant cultivars, albeit at a higher cost for farmers. Monitoring resistance-breaking strains, seed health, and the practice of obtaining seeds and tubers through informal seed systems (i.e., produced on-farm, acquired from neighbors, or sourced from local markets) poses ongoing challenges. Other farming practices that challenge efforts in disease management involve the exchange of vegetative planting materials. In Barbados, the exchange of sweet potato planting material (i.e., slips) among farmers is a common practice. It is suggested among industry officials that the exchange of potentially diseased planting material among farmers has contributed to the spread of viral infections [220]. Moreover, farmers prefer some yellow-fleshed cultivars, such as ‘Jewel’, because of their high yields and increased anthocyanin content compared to traditional white- or cream-fleshed varieties in the Caribbean, despite their susceptibility to viral infections under intense commercial cultivation [9,220]. Coupled with the presence of numerous wild *Ipomoea* species of morning glory, which may harbor viruses, these factors may all contribute to the spread of viruses in the sweet potato crop in Barbados, particularly during the dry season when insect infestation is greatest. Similarly, farmer preferences for tomato varieties other than the recommended TYLCV-tolerant ones are observed in Trinidad. Altogether, this (i) underscores the importance of employing IDM programs with various management measures, (ii) highlights the critical role of plant quarantine at entry ports and the necessity for public awareness to discourage the illegal introduction of materials (which may carry pathogens), and (iii) raises questions about breeding strategies to minimize the selection of resistance-breaking strains.

The accurate identification of vectors species, e.g., whitefly species, is important to disease management as the species differ in their resistance to insecticides and in the efficiency of virus transmission [221]. Insecticide resistance to more than 30 active ingredients in commercial insecticide formulations has already been reported [222]. Apart from resistance, failure to control whitefly populations in the field may be due to uninformed insecticide selection and non-compliant application of insecticides, e.g., recurrent use of the same active ingredients with overlapping modes of action leads to a gradual development of resistance over subsequent generations [223]. The application of excessive doses within a given cropping season in several countries has also led to the development of insecticide resistance in *B. tabaci* [223,224]. In Trinidad and Tobago, N-{1-[(6-Chloro-3-pyridyl)methyl]-4,5-dihydroimidazol-2-yl}nitramide (imidacloprid) has been the predominant insecticide used in the field but its effectiveness has declined over each successive cropping season [225]. Ideally, insecticide applications should be based on vector population numbers and only administered when thresholds have been reached. New chemistries that permit the rotation of different classes of insecticides are needed.

Further exploration into the development of gene editing strategies towards improving host resistance to virus infection is needed in the region. Elsewhere, site-directed genome editing based on CRISPR/Cas technology has been used to introduce resistance genes (virus or plant-derived) or to silence host genes (e.g., translation initiation factors or host factors) necessary for virus replication [226,227,228]. There are also the traditional methods of developing transgenic virus resistance that rely on RNAi-based technologies for managing single or multiple virus infections. These methods involve transformation with a single virus gene, multiple virus genes, or parts thereof [229]. An important challenge in the development and distribution of any genetically modified product in the Caribbean, however, lies in adhering to standard biosafety regulations [89]. Although potential environmental risks, such as gene flow and effects on non-target organisms or off-target effects on biodiversity, are difficult to predict [230], such risks cannot be ignored in a region characterized by high levels of endemic biodiversity [192]. Most Caribbean countries are in the early stages of preparing and/or revising biosafety legislation [231]. The introduction of genetically modified products without consideration of the broader social, political, and environmental implications will invite resistance, as seen with the development of virus-resistant transgenic papaya in Jamaica.

An additional challenge to controlling viruses transmitted by insect vectors is climate change [232]. Climate change is predicted to have a detrimental impact on agriculture in the Caribbean, depending on the rate and severity of the change, together with the speed and appropriateness of agency responses [233,234]. Dash et al. [235] anticipate environmental impacts, e.g., changes in patterns of weather and cyclonic events, air surface temperatures, and water availability, on multiple components of the pathosystem *inter alia*, vector population expansion, cultivar usage and other cropping practices, soil erosion, and chemical run-off. However, a limited number of studies have examined the potential impact of climate change on diseases and pests of tropical crops. Fundamentally, understanding the agro-ecological features of traditional farming systems that have stood the test of time and have resisted and/or recovered from droughts, floods, or cyclonic events could assist in developing strategies to mitigate the effects of climate change on agriculture [236]. Ghini et al. [17] propose long-term interdisciplinary studies, preferably under international programs, to assess the effects on diseases of tropical crops. These studies would examine the host physiology, epidemiology, genetics, and evolution of host–pathogen populations in response to changing climate. Singh et al. [18] recommend expanding this approach to include investigations on the biological, ecological, and evolutionary responses of host, pathogen, and vector as a result of specifically identified aspects of climate change. The Caribbean is poised to cooperate with the international plant pathology and wider agricultural community to identify and incorporate climate-smart methods of crop production.

## Figures and Tables

**Table 1:** Viruses detected in agricultural crops grown in Barbados, Jamaica, and Trinidad.

Genus	Virus	Common Host Name	Host Species	Reported Location	Year of Detection	Method of Detection ^a^	References
Endemic and potential re-emerging threats							
*Begomovirus*	Bean golden mosaic virus	Red kidney bean	*Phaseolus vulgaris*	Jamaica	1975, 1994, 1996	Symptomatology, PCR, Sanger sequencing	[31,32,33]
*Badnavirus*	Cacao mild mosaic virus	Cacao	*Theobroma cacao*	Trinidad	1944, 1947, 2005	Symptomatology, HTS	[34,35,36]
	Cacao yellow vein-banding virus	Cacao	*Theobroma cacao*	Trinidad	1944, 1947, 2005	Symptomatology, HTS	[34,35,36]
*Closterovirus*	Citrus tristeza virus	Citrus	*Citrus* spp.	Jamaica, Trinidad	1960, 1965, 2002, 2009, 2010, 2013	Bio-indexing, ELISA, RT-PCR, Sanger sequencing	[37,38,39,40,41,42]
*Cucumovirus*	Cucumber mosaic virus	Tomato, hot pepper, sweet potato	*Solanum lycopersicum*, *Capsicum chinense*, *Ipomoea batatas*	Jamaica, Trinidad	1974, 1992, 2004, 2011	Symptomology, NCM-ELISA	[43]
*Potyvirus*	Papaya ringspot virus	Papaya	*Carica papaya*	Jamaica, Trinidad	1929, 2014	Symptomatology, ELISA, RT-PCR, Sanger sequencing	[44,45]
	Potato virus Y	Sweet pepper, scotch bonnet pepper, bird pepper, tobacco, tomato	*Capsicum annuum*, *Capsicum chinense*, *Capsicum baccatum*, *Nicotiana tabacum*, *Solanum lycopersicum*	Jamaica	1950–1951, 1961, 1976, 1979, 1996, 2010	Symptomatology; Biological indicators, transmission tests, ELISA	[46,47,48,49,50,51]
*Tobamovirus*	Tobacco mosaic virus	Tomato, hot pepper	*Solanum lycopersicum*, *Capsicum chinense*	Trinidad	1974, 1992, 2006	Symptomology, ELISA	[43]
Recent and ongoing threats							
*Begomovirus*	Cabbage leaf curl virus	Cabbage	*Brassica oleracea*	Jamaica	1990s	PCR & Sanger sequencing	[52,53]
	Pepper huasteco yellow vein virus	Sweet pepper	*Capsicum annuum*	Trinidad	1995	Hybridization, PCR, Sanger sequencing	[54]
	Potato yellow mosaic virus	Tomato, sweet pepper, wild chili pepper, ochro, red kidney bean	*Solanum lycopersicum*, *Capsicum annuum*, *Capsicum frutescens*, *Abelmoschus esculentus*, *Phaseolus vulgaris*	Trinidad	1995	Hybridization, PCR, Sanger sequencing	[54]
	Tomato mottle virus	Tomato	*Solanum lycopersicum*	Trinidad	1998	Dot blot hybridization, PCR	[43]
	Tomato mosaic Havana virus	Tomato, scotch bonnet pepper	*Solanum lycopersicum*, *Capsicum chinense*	Jamaica	1994, 1996	PCR, RFLP, Sanger sequencing	[55,56]
	Tomato yellow leaf curl virus	Tomato, sweet pepper, wild chili pepper, ochro, red kidney bean, cowpea	*Solanum lycopersicum*, *Capsicum annuum*, *Capsicum frutescens*, *Abelmoschus esculentus*, *Phaseolus vulgaris*, *Vigna unguiculata*	Barbados, Jamaica, Trinidad	1993, 1994, 1995, 1998, 2014–2016	Hybridization, PCR, RFLP, Sanger sequencing	[54,55,56,57,58,59]
	Tomato yellow mosaic virus	Sweet pepper	*Capsicum annuum*	Trinidad	1995	Hybridization, PCR, Sanger sequencing	[54]
	Sweet potato leaf curl virus	Sweet potato	*Ipomoea batatas*	Barbados, Jamaica	2004, 2013, 2017, 2018	RT-PCR, Sanger sequencing, RCA, HTS	[60,61,62,63]
*Crinivirus*	Sweet potato chlorotic stunt virus	Sweet potato	*Ipomoea batatas*	Barbados, Jamaica	2001, 2013–2014	NCM-ELISA, RT-PCR, HTS	[63,64,65]
*Ipomovirus*	Sweet potato mild mottle virus	Sweet potato	*Ipomoea batatas*	Jamaica	2011	NCM-ELISA	[60,61]
*Potyvirus*	Ipomoea vein mosaic virus (Sweet potato virus 2)	Sweet potato	*Ipomoea batatas*	Barbados	2001	NCM-ELISA	[65]
	Sweet potato feathery mottle virus	Sweet potato	*Ipomoea batatas*	Barbados, Jamaica,	2001, 2013–2014	NCM-ELISA, RT-PCR, HTS	[63,64,65]
	Sweet potato latent virus	Sweet potato	*Ipomoea batatas*	Jamaica	2011	NCM-ELISA	[60,61]
	Sweet potato mild speckling virus	Sweet potato	*Ipomoea batatas*	Jamaica	2006, 2011	NCM-ELISA	[60,64]
	Sweet potato virus G	Sweet potato	*Ipomoea batatas*	Barbados, Jamaica	2001, 2006, 2013	NCM-ELISA, HTS	[61,64,65]
	Tobacco etch virus	Sweet pepper, scotch bonnet pepper, tomato	*Capsicum annuum*, *Capsicum chinense*, *Solanum lycopersicum*	Barbados, Jamaica, Trinidad	Early to mid-1990s, 2006	Symptomatology, biological indicators, transmission tests, ELISA, RT-PCR, Sanger sequencing	[43,50,51]
	Watermelon mosaic virus/papaya ringspot virus—watermelon type	Cantaloupe, cucumber, pumpkin, watermelon	*Cucumis melo*, *Cucumis sativa*, *Cucurbita pepo*, *Citrullus lanatus*	Jamaica, Trinidad	1986, 2020	Symptomatology, biological indicators transmission tests	[43,66]
Newly recognized threats							
*Begomovirus*	Ipomoea yellow vein virus	Sweet potato	*Ipomoea batatas*	Barbados	2018	RT-PCR, HTS	[65]
*Comovirus*	Squash mosaic virus (Comovirus curcubitae)	Pumpkin	*Cucurbita* spp.	Jamaica, Trinidad	2014, 2015	ELISA, RT-PCR	[67,68]
*Crinivirus*	Cucurbit yellow stunting disorder virus	Cantaloupe, cucumber, watermelon	*Cucumis melo*, *Cucumis sativa*, *Citrullus lanatus*	Jamaica	2019, 2020	RT-PCR	[69]
*Potyvirus*	Yam mild mosaic virus	Yam	*Dioscorea* spp.	Barbados	2018	RT-PCR	[43]
	Zucchini yellow mosaic virus	Pumpkin	*Cucurbita* spp.	Jamaica, Trinidad	2014, 2016	Symptomology, RT-PCR, Sanger sequencing	[67,68]

^a^ ELISA enzyme-linked immunosorbent assay; HTS high-throughput sequencing; NCM-ELISA nitrocellulose membrane enzyme-linked immunosorbent assay; PCR polymerase chain reaction; RCA rolling circle amplification; RFLP restriction fragment length polymorphism; RT-PCR reverse transcription polymerase chain reaction.

**Table 2:** Virus detections based on symptomatology, with no recent confirmations.

Genus	Virus	Common Host Name	Host Species	Reported Location	Year of Detection	References
*Alphanucleorhabdovirus*	Maize mosaic virus (Alphanucleorhabdovirus maydis)	Corn	*Zea mays*	Trinidad	1933	[43]
*Caulimovirus*	Cauliflower mosaic virus	Crucifers	*Brassica* spp.	Trinidad	1948	[43]
*Comovirus*	Cowpea severe mosaic virus	Cowpea	*Vigna unguiculata*	Trinidad	1962, 2007	[43]
*Polerovirus*	Potato leafroll virus	Tomato	*Solanum lycopersicum*	Trinidad	1974	[43]
*Potexvirus*	Cassava common mosaic virus	Cassava	*Manihot esculenta*	Trinidad	1989	[46]
*Potyvirus*	Bean common mosaic virus	Red kidney bean	*Phaseolus vulgaris*	Barbados, Jamaica	1990s	[46]
	Dasheen mosaic virus	Dasheen	*Colocasia esculenta*	Trinidad	2014	[43]
	Johnsongrass mosaic virus	Sugarcane	*Saccharum officinarum*	Jamaica	1919	[46]
	Maize dwarf mosaic virus	Sugarcane	*Saccharum officinarum*	Jamaica	1919	[46]
	Pepper vein banding virus	Sweet pepper	*Capsicum annuum*	Jamaica	1960	[46]
	Potato virus Y in rugose mosaic	Irish potato	*Solanum tuberosum*	Jamaica	1974	[193]
	Sorghum mosaic virus	Sugarcane	*Saccharum officinarum*	Jamaica	1919	[46]
	Soybean mosaic virus	Soybean	*Glycine max*	Jamaica	1940	[46]
	Sugarcane mosaic virus	Sugarcane, corn	*Saccharum officinarum*, *Zea mays*	Jamaica, Trinidad	1919, 1942, 1989, 2022	[43,46]
	Yam mosaic virus	Yam	*Dioscorea* spp.	Jamaica, Trinidad	1963, 1979	[43,46]
*Tobamovirus*	Pepper mild mottle virus	Scotch bonnet pepper	*Capsicum chinense*	Trinidad	2014	[40]

## Data Availability

Not applicable.
